# Where do those data go? Reuse of screening results from clinical trials to estimate population prevalence of HBV infection in adults in Kilifi, Kenya

**DOI:** 10.1016/j.jve.2023.100355

**Published:** 2023-12-02

**Authors:** Louise O. Downs, Cori Campbell, Michael Abouyannis, Mark Otiende, Melissa Kapulu, Christina W. Obiero, Mainga Hamaluba, Caroline Ngetsa, Monique I. Andersson, George Githinji, George Warimwe, Kathy Baisley, J. Anthony G. Scott, Philippa C. Matthews, Anthony Etyang

**Affiliations:** a Nuffield Department of Medicine, University of Oxford, Oxford, OX1 3AZ, UK; b KEMRI-Wellcome Trust Research Programme, PO Box 230, Hospital Road, 80108, Kilifi, Kenya; c Liverpool School of Tropical Medicine, Pembroke Pl, Liverpool, L3 5QA, UK; d Oxford University Hospitals, Headley Way, Oxford, OX3 9DU, UK; e Radcliffe Department of Medicine, University of Oxford, Oxford, OX1 3AZ, UK; f Department of Biochemistry and Biotechnology, Pwani University, Kenya; g Department of Infectious Disease Epidemiology, London School of Hygiene and Tropical Medicine, London, WC1F 7HT, UK; h The Francis Crick Institute, 1 Midland Road, London, NW1 1AT, UK; i Division of Infection and Immunity, University College London, London, UK; j University College London Hospital, 235 Euston Road, London, NW1 2BU, UK

**Keywords:** Chronic hepatitis B, Clinical trials, Seroprevalence, Elimination goals, Clinical care

## Abstract

Chronic hepatitis B infection (CHB) is a significant problem worldwide with around 300 million people infected. Ambitious goals have been set towards its elimination as a public health threat by 2030. However, accurate seroprevalence estimates in many countries are lacking or fail to provide representative population estimates, particularly in the WHO African Region (AFRO). This means the full extent of HBV infection is not well described, leading to a lack of investment in diagnostics, treatment and disease prevention. Clinical trials in the WHO AFRO region have been increasing over time and many test for infectious diseases including hepatitis B virus (HBV) to determine baseline eligibility for participants, however these screening data are not reported. Here we review data from six clinical trials completed at the KEMRI-Wellcome Trust Research Programme between 2016 and 2023 that screened for HBV using hepatitis B surface antigen (HBsAg) as part of the trial exclusion criteria. 1727 people had HBsAg results available, of which 60 tested positive. We generated a crude period HBV prevalence estimate of 3.5% (95% CI 2.6–4.5%), and after standardisation for sex and age to account for the population structure of the Kilifi Health Demographics Surveillance System (KHDSS), the prevalence estimate increased to 5.0% (95% CI 3.4–6.6%). The underrepresentation of women in these trials was striking with 1263/1641 (77%) of participants being male. Alanine aminotransferase (ALT) was significantly higher in the HBsAg positive group but was not outside the normal range. We argue that routine collation and publishing of data from clinical trials could increase precision and geographical representation of global HBV prevalence estimates, enabling evidence-based provision of clinical care pathways and public health interventions to support progress towards global elimination targets. We do acknowledge when using clinical trials data for seroprevalence estimates, that local population structure data is necessary to allow standardisation of results, and the point of care tests used here are limited in sensitivity and specificity.

## 1 Introduction

The number of clinical trials conducted in the WHO Africa Region has increased over time^1^ and many involve screening for infections of public health importance as part of determining eligibility for participation. One such infection widely tested for is hepatitis B virus (HBV). However, as people testing positive for HBV are typically excluded from trial participation at screening, these data are not routinely presented as part of the study output and not accessible in published data sets for reuse, collation or analysis. We therefore postulate that screening data from clinical trials may be an important untapped source of information about HBV prevalence in African populations.

Around 300 million people worldwide are chronically infected with HBV, leading to >500,000 deaths annually.^2^ Studies estimating chronic HBV (CHB) prevalence in Africa most often focus on key populations with underlying risk factors, such as people living with HIV infection, commercial sex workers or people who inject drugs, and thus do not represent the general population epidemiology.^3^ Some studies have screened blood donors, but these exclude key populations and people living with other blood-borne virus infections, so are also not representative of diverse populations. In Kenya there has been only one study since 2007 undertaking a general population HBV screening as a national survey,^4^ and even this has excluded those with HIV infection. Furthermore, most studies do not account for age and sex structure of the population, which can influence the accuracy of overall prevalence estimates. HBsAg prevalence estimates are therefore likely to be biased, particularly in the WHO Africa Region.^3^ This lack of robust data contributes to the neglect of funding, advocacy, public health interventions and clinical care pathways for CHB.

The Kenya Medical Research Institution (KEMRI) and its collaborators generate significant research outputs, including clinical trial data. We therefore set out to collate data from studies previously conducted at the KEMRI-Wellcome Trust Research Programme (KWTRP), Kilifi, that screened volunteers for HBsAg. We aimed to i) determine the scale of the potential available data source from clinical studies, and ii) derive both a crude and age/sex standardised HBsAg prevalence estimate from existing KWTRP clinical trials data.

## 2 Methods

### 2.1 Eligible studies

We determined which studies at KWTRP (enrolling adult participants (≥18 years) since January 2010, and completed by January 2023 had screened for HBsAg (± measured alanine aminotransferase, ALT levels)), through review of laboratory data generated by the local Research Departments and Laboratories over this time. HBsAg data was only accessible from 2016 onwards as prior to this routine HBsAg testing was not widely available through the central laboratory at KWTRP.

### 2.2 Study populations

All study screening was done in community groups from the Kilifi Health and Demographic Surveillance System (KHDSS), other than the SERU 4024 which recruited frontline staff (Supplementary Table 1). All studies screened healthy adults (age ≥18 years) with no known chronic disease prior to initial testing. Studies typically restricted the ages of screened participants, with only two studies allowing a broad range of ages from 18 to 64 years. All studies excluded pregnant or breastfeeding women, along with any women planning to become pregnant during the trial. Women were also required to take birth control during the trial; in most cases female participants were referred to a local family planning clinic.

In addition to the data from KWTRP, we also searched Clinicaltrials.gov for other completed vaccine trials conducted in Kenya since 2010 that had recruited adults aged ≥18 years and screened for HBsAg prior to enrolment to estimate the amount of data that may be available over a larger geographical area.

### 2.3 Ethics and governance

Ethical approval from both the Scientific Ethics Review Unit (SERU) in Kenya and the Oxford Tropical Research Ethics Committee (OxTREC) for the collection and collation of existing KWTRP data was obtained (SERU approval number 4565, OxTREC approval number 22–23). Principal investigators for eligible studies were asked to approve data use, and then an application was submitted to the KWTRP data governance committee for data access. When necessary, permission from study sponsors was also sought.

### 2.4 Data collection

Data managers were contacted to provide study protocols to identify characteristics of the target study population. Data on ALT and HBsAg from the initial trial screening were extracted from the Kilifi Integrated Data Management System (KIDMS) at KWTRP and demographic data including age at time of screening and sex for each individual participant were extracted from the study database. All data collected were anonymised.

### 2.5 Generation of laboratory data

For these studies, Ilab Aries (Instrumentation Laboratory, USA) had been used for alanine transferase (ALT) measurement and point of care HBsAg testing kits were used for HBsAg screening (QuickProfile HBsAg test (Lumiquick Diagnostics Inc, USA) or Hexagon HBsAg test (Human Diagnostics Worldwide, Wiesbaden, Germany)). The upper limit of normal (ULN) for ALT in the Kilifi population is 35 U/L for women and 55 U/L for men (2021 locally derived reference ranges). Where electronic data were not available, paper copies of patient screening forms were reviewed.

### 2.6 Statistical analysis

Overall and age-sex stratified crude HBsAg prevalence estimates were calculated for the period covered by the studies, from 2016 to 2022. We adjusted for bias in the age-sex structure of the clinical trial samples by calculating age-sex standardized estimates using the 2019 KHDSS data as the reference population.^5^ Wilcoxon rank sum test was used to analyse differences in ALT between different sexes and those who were HBsAg positive and negative. Analyses were conducted using R (version 4.2.0) and STATA/IC version 15.1 (StataCorp College Station, Texas, USA, RRID:SCR_012763).

## 3 Results

### 3.1 KWTRP study data

**Supplementary Fig. 1:**
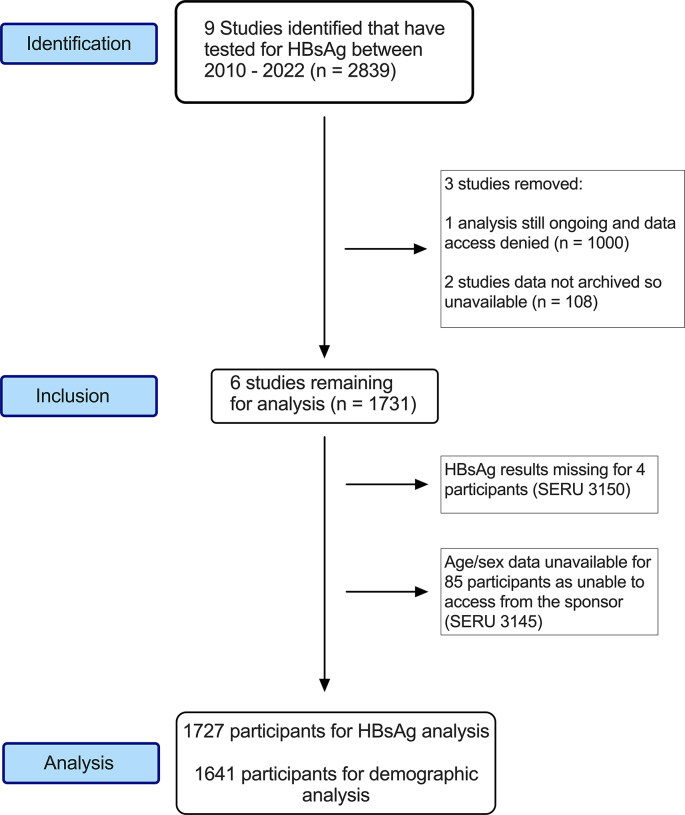
STROBE diagram indicating studies identified which screened for HBsAg at KWTRP between 2010 and 2023, detailing reasons for exclusion and numbers of participants included in the analysis.

We identified nine studies which tested potential participants for HBsAg, representing 2839 individuals. Of these studies, 3 were excluded due to data being unavailable (n = 1108) leaving six studies representing 1727 participants (Supplementary Fig. 1). 6, 7, 8, 9, 10, 11 1641 individuals had age and sex data recorded alongside HBsAg. Most participants were male (1263/1641, 77%) and median age was 28 years (IQR 23–36 years). Study recruitment and characteristics are shown in Supplementary Table 1.

### 3.2 HBsAg prevalence

Of 1727 individuals tested, 60 were HBsAg positive, giving a crude period prevalence estimate of 3.5% (95% CI 2.6–4.5%, prevalence range between studies 1.9 %–3.9%). Standardised HBsAg positivity was highest in those aged >55 years (6.3% CI 2.9–9.7, Table 1). Crude prevalence was higher in men compared with women (3.9% vs 2.1% respectively, Table 1), however when standardised, confidence intervals for HBsAg prevalence in women were too wide to make any conclusions. Overall standardised HBsAg population prevalence was estimated at 5.0% (CI 3.4–6.6), still higher than the 3.5% crude prevalence when only including the 1641 participants for which age and sex data were available.

**Table 1:** Crude and standardised hepatitis B surface antigen (HBsAg) prevalence rates derived from six clinical trials and standardised by age and sex based on the structure of the Kilifi Health and Demographic Surveillance System (KHDSS). CI = Confidence Interval.

Group	Total Tested	Proportion of the tested population	Crude HBsAg Seroprevalence (%)	Total in KHDSS population	Proportion of the KHDSS population	Standardised HBsAg Prevalence (%)	95 % CI of the standardised prevalence
**Overall**							
**Crude**	1727		3.5				
**Standardised**	1641		3.5	139303		5.0	3.4–6.6
**Sex**							
**Male**	1263	1263/1641	3.9	61006	61006/139303	3.3	2.4–4.2
**Female**	378	378/1641	2.1	78298	78298/139303	5.0	0.0–14.2
**Age Group in Years**							
**18**–**25**	632	632/1641	3.6	40624	40624/139303	2.2	1.0–3.4
**26**–**35**	588	588/1641	2.4	36686	36686/139303	2.1	0.9–3.4
**36**–**45**	338	338/1641	5.6	26314	26314/139303	4.3	1.7–6.9
**46**–**55**	65	65/1641	0	16732	16732/139303	0.0	NA
**>55**	18	18/1641	5.6	18946	18946/139303	6.3	2.9–9.7

### 3.3 ALT data

**Fig. 1:**
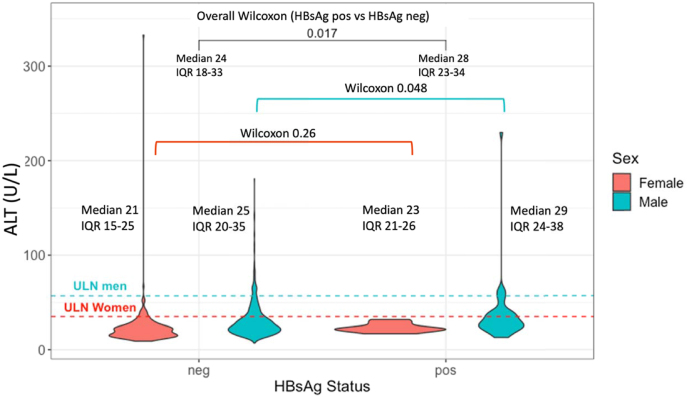
Violin plots showing distribution of alanine transaminase (ALT) in adults by HBsAg status and sex in studies conducted at the KEMRI-Wellcome Trust Research Programme. ALT reference ranges are shown with dashed horizontal lines showing upper limit of normal (ULN) based on 2021 thresholds derived locally at KWTRP (male ULN = 57 U/L, women ULN = 35 U/L). Wilcoxon p-values are shown comparing ALT in those who were HBsAg positive and negative within each sex and overall between all those who were HBsAg positive and negative.

Of the 1727 participants screened for HBsAg, 1462 also had ALT measured (85 %). ALT ranged from 7 to 333 U/L, with a median of 24 U/L (IQR 19–33 U/L). Median ALT was higher in those who were HBsAg positive (24 vs 28 U/L, Wilcoxon rank sum p = 0.017), although was not over the ULN for either group. This median difference in ALT between HBsAg positive and negative adults was significant among males but not females (Fig. 1).

### 3.4 Data from Clinicaltrials.gov

We identified ten studies from Clinicaltrials.gov in addition to those from above, that had screened for HBsAg, enrolling a total of 1253 participants. Based on our assessment of KWTRP studies, the ratio tends to be at least 2 people screened for every person enrolled to a study (although this does vary between studies), indicating there could be potential information on HBsAg status from around another 2500 people in Kenya if data from other studies on Clinicaltrials.gov could be accessed.

## 4 Discussion

We have demonstrated that there are valuable HBsAg prevalence data in Kilifi, Kenya that have not been previously collated or reported. Studies currently ongoing will, in due course, collect data for a further >1000 participants, and existing data on Clinicaltrials.gov has the potential to further expand and broaden the geographical range of available data. The dataset of >1700 adults we have collated is comparable in size to the largest pre-existing HBsAg seroprevalence studies in Kenya (reviewed in Ref. ^12^), and although the crude seroprevalence estimates here are similar to those in the national survey or previous studies screening blood donors,^4^^,^^13^^,^^14^ the standardised estimate is considerably higher. The populations included in the studies reviewed here were not aimed to be representative of the KHDSS population structure or designed to assess HBsAg seroprevalence. Standardization by age and sex is therefore necessary to obtain an estimate that is representative of the KHDSS population and will also be required when using other clinical trials data to estimate disease seroprevalence.

Large seroprevalence studies are expensive and require substantial infrastructure, and resources to deliver. In contrast, the results we have generated here capitalise on the use of pre-existing data, with relatively low additional expenditure required, although time and effort are needed to manage data governance, collation and analysis.

HBsAg status was associated with a small but significant elevation in ALT, suggesting HBV infection as a driver of liver inflammation in this population, highlighting the opportunity to identify those with CHB infection for closer monitoring and treatment. This is in line with previous studies in East Africa identifying CHB as a significant cause of deranged LFTs in different populations.^15^^,^^16^

The male excess in clinical trials shown here is striking and highlights the underrepresentation of women. All trials had strict exclusion criteria around pregnancy and excluded women not receiving contraception for the trial duration. Given the age criteria of many of these studies, most eligible women were likely to be of childbearing age, leading to exclusion even from screening. It is also possible that fewer women presented for screening due to other responsibilities such as work, childcare and household management preventing trial participation. The need to include pregnant and breastfeeding women in clinical trials has previously been highlighted,^17^^,^^18^ and although clearly there should be some stratification regarding inclusion of this group, broad exclusion of all women of reproductive age leaves clinicians and women with little or no high-quality data on which to base intervention decisions.

## 5 Limitations

Individuals who volunteer to take part in clinical trials are not completely representative of the general population, being a group who are usually healthier, more educated and engaged in research. HBV prevalence is likely to be lower in this group than in the general population. When studies are discussed with community groups to invite study volunteers, exclusion criteria will be made clear. Therefore those known to have HBV or liver disease may chose not to attend screening, leading to a lower HBV population prevalence estimate. When using clinical trials more widely for prevalence estimates, the population included in the trial must be considered prior to assessment of disease prevalence and any application to a more general population.

Point of care tests (POCT) were used in these trials to screen for HBsAg. These are not as accurate as laboratory-based enzyme-linked immunoassay (ELISA) and this should be considered when interpreting results. Published specificity of these tests varies from 97% for Hexagon to 99.5% for QuickProfile,^19^^,^^20^ so some positive results here are likely to be false. Sensitivity is however also variable, so there will be some false negative cases. Newer POC HBsAg tests have improved sensitivity and specificity to widely be >99.5%. False positive results will therefore be less of a concern when using more recent clinical trials data.

The ALT measurement and HBsAg testing were done at the same visit and no longitudinal ALT data was reviewed to evaluate longer term liver function. Many other factors can influence ALT (such as medications, alcohol, aflatoxin consumption, a range of acute and chronic infections) and these may contribute to any derangement at a single time point.

Triple HBV vaccination at birth was introduced into Kenya in 2001 as part of the routine childhood immunisation programme, so those born after this date are likely to have a lower HBV seroprevalence than older adults who were born prior to widespread vaccination. This should be born in mind when applying this prevalence estimate to older age groups. So few women were included in these studies that useful estimates of HBsAg prevalence in women are not possible, even when standardised by sex.

## 6 Conclusion

Here we have used existing clinical trials data to calculate HBV seroprevalence in Kilifi, Kenya, standardised by age and sex. This estimate is higher than previous figures but likely to be representative of the general Kilifi population due to age-sex standardization to the local population structure. This estimate has been obtained without any extra laboratory or fieldwork and is therefore a highly efficient surveillance exercise, providing a prevalence estimate in a setting where such data are relatively rare and often poorly representative of the general population. We show a small significant increase in ALT in those who are HBsAg positive, and also highlight the under-representation of women in clinical trials. The secondary value of clinical trials screening data is sufficiently high to justify the archiving of all hepatitis serology and liver enzyme data, and more broadly hepatitis C and HIV data. This could then provide the substrate for modelling not only HBsAg prevalence by age, sex and geography, and estimating the associated burden of liver disease, across the WHO African region, but also prevalence of other chronic blood borne viral infections.

## Author contributions

Study concept and manuscript writing: Louise Downs, Philippa Matthews, Monique Andersson, Anthony Scott, Anthony Etyang.

Provided study data: Anthony Scott, Mainga Hamaluba, George Warimwe, Melissa Kalpulu, Mike Abouyannis, Christina Obiero, Caroline Ngetsa.

Data analysis and interpretation: Louise Downs, Cori Campbell, Mark Oteinde, Kathy Baisley, Anthony Scott, Monique Andersson, Philippa Matthews, Anthony Etyang.

Writing Assistance: Philippa Matthews, Monique Andersson, Anthony Scott, Anthony Etyang.

Proof reading article: All Authors.

## Funding

This research was funded in whole or in part by the 10.13039/100010269Wellcome Trust [Grant number 225485/Z/22/Z], For the purpose of Open Access, the author has applied a CC-BY public copyright license to any author accepted manuscript version arising from this submission. LOD is funded by a 10.13039/100010269Wellcome Trust Grant (number 225485/Z/22/Z) and Oxford University John Fell Fund (award number 0012112). PCM is supported by core funding from the 10.13039/100010438Francis Crick Institute, by a 10.13039/100004440Wellcome fellowship (ref 110110/Z/15/Z), and University College London NIHR Biomedical Research Centre (BRC). MAb is supported by a 10.13039/100010269Wellcome Trust fellowship: 203919/Z/16/Z. The funders had no role in undertaking this review.

## Declaration of competing interest

The authors declare that they have no known competing financial interests or personal relationships that could have appeared to influence the work reported in this paper.

## Data Availability

Data will be made available on request.
