# Supplementary material for: Where do those data go? Reuse of screening results from clinical trials to estimate population prevalence of HBV infection in adults in Kilifi, Kenya
Source: J Virus Erad. 2023 Dec 2;9(4):100355. doi: 10.1016/j.jve.2023.100355 (PMC10783622; doi:10.1016/j.jve.2023.100355)
Supplement: Multimedia component 1 [file mmc1.docx]

|  | **SERU study number, topic and phase (where applicable)** | **Age restrictions** | **Recruitment dates** | **N (adults) screened for HBsAg** | **Groups excluded** | **Target population**  **Recruitment strategy** |
| --- | --- | --- | --- | --- | --- | --- |
| 1 | SERU 4024. Covid vaccine study.  Phase 1b/2. | 18-64 | 2020 - 2022 | 680 | Pregnant women  Bf women  Those planning pregnancy | Healthy adults - Frontline staff as defined by the government of Kenya*.  Recruited through posters advertising the study – individuals had to get in touch with study team. |
| 2 | SERU 4106. Use of Unithiol for snakebite.  Phase 1 (5). | 18-64 | 2019 - 2023 | 156 | Pregnant women  Bf women | Healthy adult volunteers recruited from the KHDSS using established strategies e.g. health talks, barazas, community health volunteers and fieldworkers |
| 3 | SERU 3150. Shigella vaccine study (6).  Phase 2a | 18-45 | 2016 - 2017 | 132 | Pregnant women  Bf women  Those planning pregnancy | Healthy adult volunteers recruited from Kilifi town. |
| 4 | SERU 3190. Malaria inoculation study (7) | 18-45 | 2016 - 2020 | 638 | Pregnant women  Bf women  Those planning pregnancy | Healthy adult volunteers** living in Ahero, Kilifi North and South locations with high, low and moderate malaria transmission respectively through community wide meetings. |
| 5 | SERU 3711. Malaria vaccine study (8).  Phase 1b | 18-45 | 2018 - 2022 | 40 | Pregnant women  Bf women  Those planning pregnancy | Healthy adult volunteers recruited from the whole KHDSS using established strategies e.g. health talks, barazas, community health volunteers and fieldworkers |
| 6 | SERU 3145  *Streptococcus pneumoniae* Vaccine study (9)  Phase 1/2 | 18-40 | 2016 - 2018 | 85 | Pregnant women  Bf women  Those planning pregnancy | Healthy adult volunteers recruited from the whole KHDSS using established strategies e.g. health talks, barazas, community health volunteers and fieldworkers |

**Supplementary Table 1:** Characteristics of studies previously conducted at KWTRP which screened potential participants for HBsAg. Bf = Breastfeeding. SERU = Scientific ethics review unit. References given are for the study protocol, study location on ClinicalTrials.gov, or a completed study manuscript.

*Frontline staff included healthcare workers, allied health professionals, truckers, security personnel, banking personnel, supermarket staff, police, security personnel, prison workers, laboratory technicians, scientists, logistics personnel, public transport workers including aviation industry amongst others.

KHDSS – Kilifi Health Demographic Surveillance System. **Of the 638 volunteers, 134 volunteers were repeat screens over a period of more than 120 days.
